# Use of inhaled corticosteroids in preschool children and variability among pediatricians: a real-world analysis before and during the SARS-CoV-2 pandemic

**DOI:** 10.1186/s12887-023-03968-5

**Published:** 2023-04-01

**Authors:** Michela Alagna, Antonio Clavenna, Laura Reali, Adele Lallo, Danilo Fusco, Marina Davoli, Mirko Di Martino

**Affiliations:** 1 Department of Epidemiology, Lazio Regional Health Service, Via Cristoforo Colombo, Rome, 112 – 00147 Italy; 2 Laboratory of Epidemiology of Developing Age, Department of Medical Epidemiology, Istituto di Ricerche Farmacologiche Mario Negri IRCCS, Milan, Italy; 3 Primary care pediatrician, ASL Roma 1, Rome, Italy; 4 Regional Directorate for Health and Social Policy, Lazio Region, Rome, Italy

**Keywords:** Inhaled corticosteroid prescribing, Preschool children, SARS-CoV-2 pandemic, Variability in prescribing habits, Multilevel models

## Abstract

**Background:**

In Italy, inhaled corticosteroids (ICSs) are inappropriately prescribed to provide relief in URTI symptoms. Extreme variation in ICS prescribing has been described at regional and sub-regional level. During 2020, extraordinary containment measures were implemented in attempt to halt Coronavirus, such as social distancing, lockdown, and the use of mask. Our objectives were to evaluate the indirect impact of the SARS-CoV-2 pandemic on prescribing patterns of ICSs in preschool children and to estimate the prescribing variability among pediatricians before and during the pandemic.

**Methods:**

In this real-world study, we enrolled all children residing in the Lazio region (Italy), aged 5 years or less during the period 2017–2020. The main outcome measures were the annual ICS prescription prevalence, and the variability in ICS prescribing, for each study year. Variability was expressed as Median Odds Ratios (MORs). If the MOR is 1.00, there is no variation between clusters (e.g., pediatricians). If there is considerable between-cluster variation, the MOR will be large.

**Results:**

The study population consisted of 210,996 children, cared by 738 pediatricians located in the 46 local health districts (LHDs). Before the pandemic, the percentage of children exposed to ICS was almost stable, ranging from 27.3 to 29.1%. During the SARS-CoV-2 pandemic, the ICS prescription prevalence dropped to 17.0% (p < 0.001). In each study year, a relevant (p < 0.001) variability was detected among both LHDs and pediatricians working in the same LHD. However, the variability among individual pediatricians was always higher. In 2020, the MOR among pediatricians was 1.77 (95% CI: 1.71–1.83) whereas the MOR among LHDs was 1.29 (1.21–1.40). Furthermore, MORs remained stable over time, and no differences were detected in ICS prescription variability before and after pandemic outbreak.

**Conclusions:**

If on one hand the SARS-CoV-2 pandemic indirectly caused the reduction in ICS prescriptions, on the other the variability in ICS prescribing habits among both LHDs and pediatricians remained stable over the whole study time span (2017–2020), showing no differences between pre- pandemic and pandemic periods. The intra-regional drug prescribing variability underlines the lack of shared guidelines for appropriate ICS therapy in preschool children, and raises equity issues in access to optimal care.

**Supplementary Information:**

The online version contains supplementary material available at 10.1186/s12887-023-03968-5.

## Background

The prescribing pattern of drugs in the Italian pediatric population is characterized by a greater prescription of anti-asthmatic drugs. More specifically, in 2019 anti-asthmatic drugs, and inhaled corticosteroids (ICSs) in particular, were the second most commonly prescribed therapeutic class after antibiotics [1]. In children without a diagnosis of asthma the ICS prevalence rate was more than 5-fold higher than in UK and the Netherlands [2]. In Italy, ICSs are mainly prescribed to children aged 0–5 as nebulized suspension for the control of symptoms of upper respiratory tract infections (URTIs), such as cough, pharyngotonsillitis, rhinitis, otitis media, and sinusitis [3, 4], despite there is no evidence of their effectiveness.

In a trial involving 520 preschool children with URTIs there was no difference in the timing of symptoms disappearance between subjects exposed to beclomethasone and subjects exposed to placebo. Additionally, therapy with cortisone did not significantly change the likelihood of subsequent pediatric visits, emergency room access and hospital admissions [5].

Moreover, extreme variation in anti-asthmatic prescribing rates has been described at regional and sub-regional level [6]. The high ICS prevalence rate and the large variability in anti-asthmatic prescribing habits raise concerns about the rational use of respiratory drugs in the Italian pediatric population.

During 2020, restrictions over the entire country were implemented in attempt to halt Coronavirus. Moreover, the extraordinary containment measures (e.g., social distancing, lockdown and the use of mask) were associated with a generalized reduction in URTIs [7–10]. No evidence exists about the impact of SARS-CoV-2 restrictions on ICS prescribing attitude and its variability among pediatricians.

### Objectives

The objectives of this study are to measure the ICS prescription prevalence in preschool outpatients of the Lazio region, to evaluate the indirect impact of the SARS-CoV-2 pandemic on the prescribing patterns of ICSs, and to estimate the prescribing variability among pediatricians before and during the SARS-CoV-2 pandemic.

## Methods

### Setting and data sources

In Italy, a central role in the governance of primary-care prescription patterns is played by the Local Health District (LHD), a body delegated by the National Health System to provide health care to a specific geographic area. Each LHD is composed of a well-defined group of primary care physicians (including general practitioners and pediatricians) sharing the same socioeconomic and cultural backgrounds, coordinated by a district director. Acting in this framework, LHDs and physicians may have a synergistic effect on variation in ICS prescription. In fact, physicians are influenced by the groups to which they belong, and the properties of those groups are in turn influenced by the physicians who make up that group. In this real-world study, subjects were recruited from individuals registered with the LHDs of the Lazio Region (about 5 million residents), who were eligible over the study period. Drug information was collected from the regional Drug Claims Register (DCR). Data available on each prescription include the patient’s identification number, the prescribing physician’s number, the Anatomical-Therapeutic-Chemical (ATC) code of the drug purchased, the number of packs, the number of units per pack, the dosage, the unit cost per pack and the prescription date. The drugs under study are equally available for all residents, in accordance with the universal health-care insurance coverage. A unique and anonymous patient identifier was used to link drug information with additional data retrieved from the information systems described below.

The *Hospital Information System* (HIS) includes socio-demographic and clinical inpatient data (e.g., date of admission and discharge, diagnoses, and procedures).

The *Emergency Information System* (EIS) collects demographic and clinical data on patient entering the emergency room (e.g., date of access and exit, triage code, and diagnoses).

The *Ticket Exemption Information System* (TEIS) contains information on ticket exemption (e.g., date of activation, pathology, and exemption code). In Italy, patient suffering from a disease for which an exemption is provided can take advantage of free drugs and services for the control and treatment of the pathology.

Finally, the *Emergency Coronavirus Information System* (ECIS) records information on all notified cases of SARS-CoV-2 infection, ascertain by positive molecular or antigen test.

### Study population and drug exposure

The main study population includes all children aged 5 years or less on December 31, 2019 (the *index date*). ICS drugs under study were those with the availability of a nebulized formulation: beclomethasone (ATC: R03BA01), budesonide (R03BA02), flunisolide (R03BA03) and fluticasone (R03BA05). ICS prescriptions were collected and analyzed during a one-year follow-up, starting from the index date. Drugs exposure was measured by the prescription prevalence, expressed as the number of children who received at least one prescription of ICS during follow-up, per 100 individuals in the population.

In order to describe and compare ICS prescribing patterns over time, the same study design was replicated considering the following *index dates*: December 31, 2016; December 31, 2017; December 31, 2018.

### Exclusion criteria

Children with asthma/recurrent wheezing were excluded from the analysis. Other exclusion criteria were cystic fibrosis, immunodeficiency, and malignant tumor, because of the complex clinical conditions and the potentially long hospital stay.

In order to allow an unbiased comparison over time, children registered in ECIS, who therefore contracted SARS-CoV-2 infection during 2020 were excluded from the analysis.

The presence of asthma, cystic fibrosis, immunodeficiency, and malignant tumor was ascertained through diagnoses from hospital discharge records or admissions to emergency room, disease-specific ticket exemptions and drug use patterns. For an operational definition of the exclusion criteria, see the Additional file 1.

### Sensitivity analysis: drug prescriptions for chronic conditions

In order to evaluate whether SARS-CoV-2 pandemic also had indirect effects on drug prescriptions for chronic conditions, a sensitivity analysis was conducted by measuring and comparing over time the proportions of children exposed to antidiabetic (ATC code: A10) and antiepileptic (ATC: N03) drugs.

### Statistical analysis

The ICS prescription prevalence was analyzed by calendar year, month, child’s gender and LHD, across the whole study time span (2017–2020). Differences in prescription prevalence were analyzed using chi-square test.

Two maps of the Lazio region were produced in order to show and compare the ICS prescription prevalence by LHD before (2017–2019) and during (2020) the SARS-CoV-2 pandemic. The classes used in the maps were calculated applying the Jenks natural breaks optimization algorithm [11], which reduces the variance within classes and maximizes the variance between classes.

Because pediatricians are nested within LHDs, the data have a hierarchical structure and cannot be considered as independent [12, 13]. In order to take into account the intraclass correlation, logistic multilevel models were performed to analyze geographic variation, by measuring and comparing the variability in ICS prescription patterns attributable to LHDs and primary care physicians. More specifically, this methodology allows to estimate two independent components: variation among LHDs and variation among pediatricians working in the same LHD (i.e., variation among pediatricians within LHDs). These variance components were calculated for each calendar year (2017–2020), and expressed in terms of Median Odds Ratio (MOR). This measure quantifies the variation among clusters and is always greater than or equal to 1.00. If the MOR is 1.00, there is no variation between clusters. If there is considerable between-cluster variation, the MOR will be large [14]. P-values and 95% confidence intervals (95% CIs) were reported. It is worth mentioning that MORs are weighted and precise variance estimators, specifically formalized for hierarchical data structures. In fact, the regression coefficients (e.g., the ICS prescription prevalences) are shrunk back towards the mean coefficient for the whole data set. The shrinkage weight depends on the reliability of the estimated coefficient. Coefficients that are estimated with small accuracy shrink more than very accurately estimated coefficients. Accuracy of estimation depends on two factors: the group sample size, and the distance between the group-based estimate and the overall estimate. Estimates for small groups are less reliable, and shrink more than estimates for large groups. Other things being equal, estimates that are very far from the overall estimate are assumed less reliable, and they shrink more than estimates that are close to the overall average [12].

## Results

**Fig. 1:**
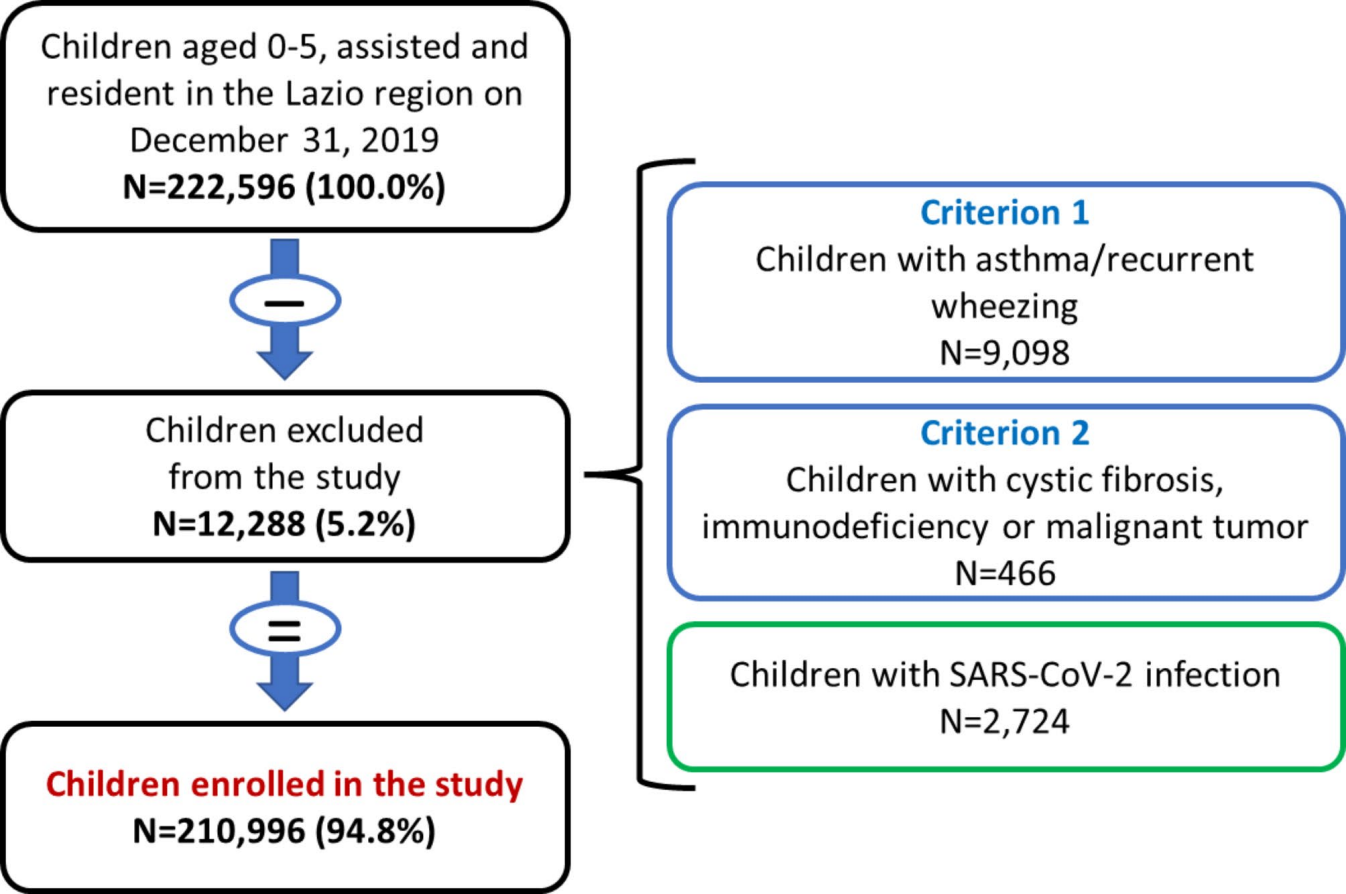
The main study population. Flow-chart

The study population consisted of 210,996 children (Fig. 1), cared by 738 pediatricians located in the 46 LHDs of the Lazio Region. Among preschool children, the prevalence of male gender was 51%.

**Fig. 2:**
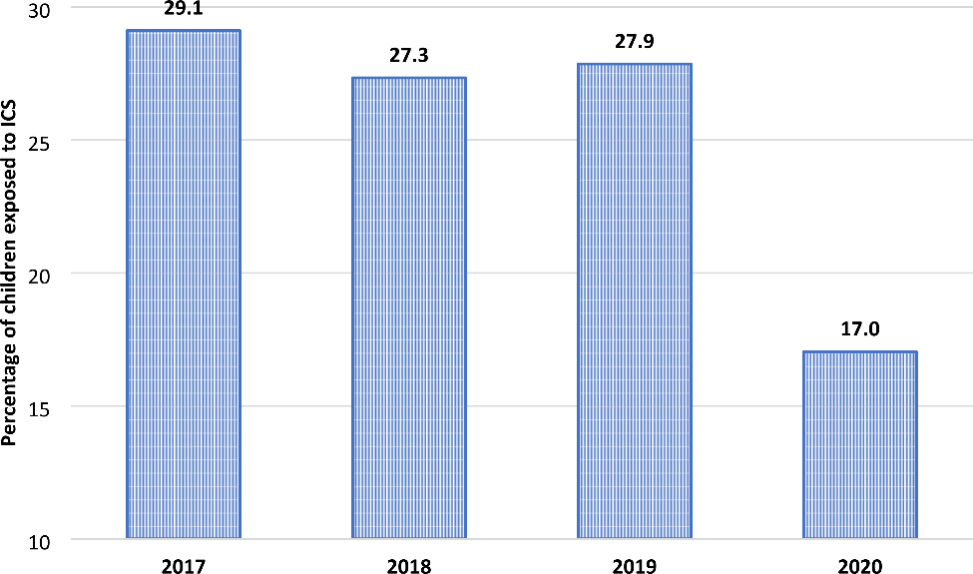
Preschool children exposed to ICS. Lazio, 2017–2020

Figure 2 shows the ICS prescription prevalence during the 2017–2020 period. Before the outbreak of pandemic (2017–2019), the percentage of children exposed to ICS was almost stable, ranging from 27.3 to 29.1%, around a mean value of 28.1%. During the SARS-CoV-2 pandemic (2020), the ICS prescription prevalence dropped to 17.0%, with a relative reduction of 39.5% (p < 0.001) with respect to the mean of the previous three years.

Results from the sensitivity analysis on drug prescriptions for chronic conditions are shown in the Additional file 2. The proportions of preschool children exposed to antidiabetic and antiepileptic drugs remained stable over the whole study period.

Returning to the main analysis, it is worth noting that females were less likely to be prescribed ICSs in each year of the study period, both before and during the pandemic. The ICS prescription prevalence (males versus females) was 30.0 vs. 28.2 in 2017 (p < 0.001) 28.2 vs. 26.4 in 2018 (p < 0.001), 28.8 vs. 26.9 in 2019 (p < 0.001), and 17.9 vs. 16.2 in 2020 (p < 0.001).

**Fig. 3:**
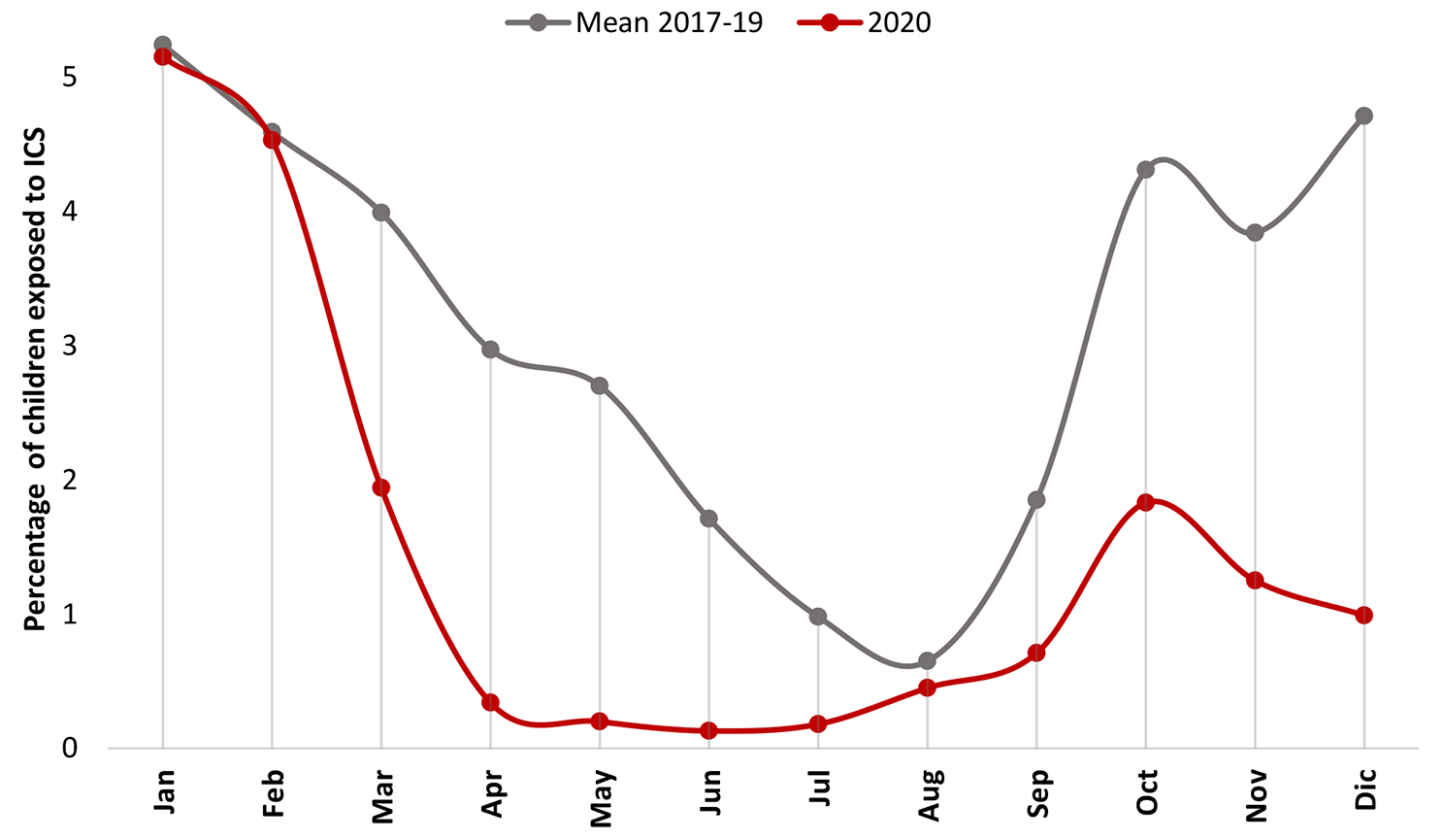
Preschool children exposed to ICS, by month. Lazio, 2017–2020

In Fig. 3, the ICS prescription prevalence was calculated by month, comparing 2020 against a counterfactual trend, obtained by averaging values from the previous three years. Immediately after the outbreak began (the first outbreak of COVID-19 in Italy occurred during the second half of February 2020), ICS prescriptions rapidly declined, showing a slight recovery in the following months. Moreover, if we consider the 2017–2019 period, the ICS prescription prevalence appears to follow a seasonal trend with higher incidence observed from October through February, peaking in December and January.

**Fig. 4:**
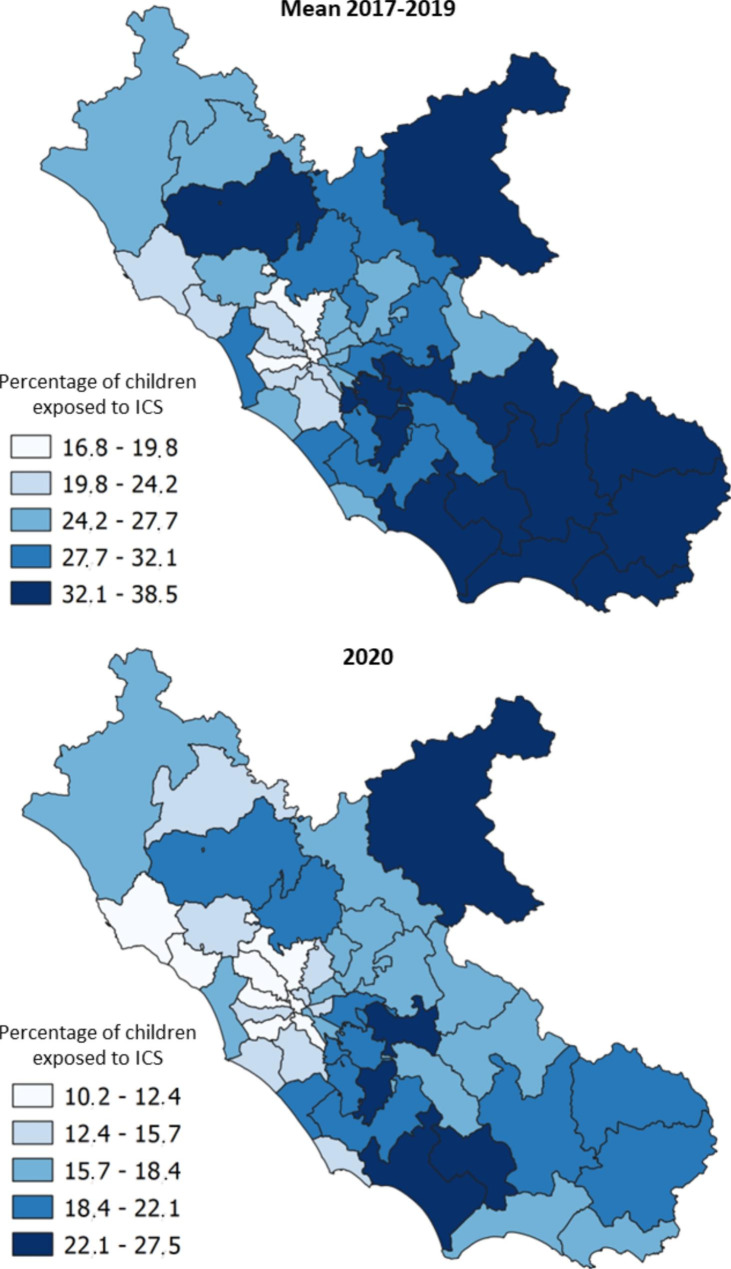
Preschool children exposed to ICS, by Local Health District. Lazio, 2017–2020

Figure 4 is composed of two maps, comparing the geographic variation in ICS prescription prevalence before (2017–2019 period) and during (2020) the SARS-CoV-2 pandemic. Before the pandemic outbreak, a high geographic variation was observed between the LHDs of the region. The percentages of children exposed to ICS ranged from 16.8 to 38.5%. During the SARS-CoV-2 pandemic the ICS prescription prevalence markedly dropped in all of the LHDs, however, the geographic variation appears substantially unchanged, with percentages ranging from 10.2 to 27.5%. With regard to variation among pediatricians, before the pandemic the ICS prescription prevalence ranged from 3.3 to 64.4%, whereas, during the pandemic from SARS-CoV-2 percentages ranged from 1.3 to 56.4%.

Results of multilevel models are presented in Table 1. For each year of the study period, variation in ICS prescription prevalence among LHDs (LHD - MOR) and among pediatricians working in the same LHD (Pediatricians - MOR) are shown. Moreover, it is possible to evaluate the trend of variation over time. In each year, a relevant and statistically significant variability was detected among both LHDs and pediatricians working in the same LHD (i.e., the variation within LHDs). However, the variability among individual pediatricians was always higher. Furthermore, the MORs remained stable over time, and no differences were detected in ICS prescription variability before and after the pandemic’s outbreak.

**Table 1:** The variability among primary care providers in ICS prescribing. Lazio, 2017–2020

Primary care provider	LHD			Pediatrician		
Year	**MOR**	p-value	95% CI	**MOR**	p-value	95% CI
2017	1.37	< 0.001	1.28–1.49	1.65	< 0.001	1.61–1.70
2018	1.28	< 0.001	1.21–1.38	1.70	< 0.001	1.65–1.75
2019	1.26	< 0.001	1.18–1.36	1.75	< 0.001	1.70–1.81
2020	1.29	< 0.001	1.21–1.40	1.77	< 0.001	1.71–1.83

Estimated Variance components (VC):

2017: LHD-VC = 0.108; Pediatrician-VC = 0.277;

2018: LHD-VC = 0.066; Pediatrician-VC = 0.308;

2019: LHD-VC = 0.057; Pediatrician-VC = 0.346;

2020: LHD-VC = 0.071; Pediatrician-VC = 0.359

## Discussion

In this study of almost 211,000 preschool children, we found a very high ICS prescription prevalence. In the 2017–2019 period, the percentage of children exposed to ICS ranged around a mean value of 28%, giving evidence that ICS prescription rates in Italian children are among the highest in Europe, as already documented in previous studies [2, 6]. During 2020, the ICS prescription prevalence significantly dropped to 17%. The analysis by month revealed that decreases in ICS prescribing started almost immediately after the pandemic began. The amount and timing of decrease in prescription rates support the hypothesis that SARS-CoV-2 outbreak indirectly led to a sharp decrease on the proportion of children exposed to ICS. In fact, it is known that, in Italy, ICSs are inappropriately prescribed to provide relief in URTI symptoms [3, 4]. Measures adopted for the control of SARS-CoV-2 pandemic were associated with a reduction in acute respiratory infections in preschool children [9, 10] and, therefore, with a decrease in ICS prescription rates.

Moreover, results from sensitivity analyses allow us to rule out the possibility that the reduced proportion of children exposed to ICS in 2020 was caused by altered care-seeking behavior or limited access to outpatient services; in fact, the prescribing prevalence of antidiabetic and antiepileptic drugs remained unchanged from the pre-pandemic period.

Statistically significant differences between males and females were observed before and during the SARS-CoV-2 pandemic: females were consistently less likely to be prescribed ICSs.

Although explanations about sex differences in ICS use are still unclear, it is likely that they are mainly due to differences in the prevalence of wheezing and URTIs [5, 15, 16], and our results are in line with previous evidence from Southern Italy and from other countries [2, 4, 17].

As regards the analysis of variance components, results of multilevel models can be very interesting from a policy perspective. First, a relevant and unwarranted geographic variation in ICS prescribing was observed among the LHDs of the Lazio region. Second, a high “within” variability was detected among pediatricians working in the same LHD.

Moreover, if on one hand the SARS-CoV-2 pandemic indirectly caused the reduction in ICS prescriptions, on the other the variation in ICS prescribing habits among both LHDs and pediatricians remained stable over the whole study time span (2017–2020), showing no differences between pre- pandemic and pandemic periods.

A high level of variation in prescribing approaches has previously been observed in Italian pediatric primary care [5, 6]. The between-provider heterogeneity in ICS prescribing raises equity concerns in access to optimal care and underlines the lack of evidence-based therapeutic protocols shared at regional level. Multilevel models showed that, in each of the four years of the study, most of the variability is attributable to the pediatrician-level. This finding supports the hypothesis that pediatricians’ characteristics, both observed and unobserved, heavily contribute to variation in usual medical care.

We don’t know why a pediatrician might make an inappropriate prescriptive choice; some inappropriate ICS prescribing may be due to a lack of awareness of evidence of their ineffectiveness in the treatment of URTIs, or lack of awareness of clinical guidelines.

Moreover, there is evidence that drug prescriptions for the treatment of respiratory infections in Italy is influenced by parental expectations: physicians are more likely to prescribe a particular drug when they perceive an expectation of such treatment from parents, although they report that parental expectations have no impact on their behavior [18].

By using administrative data, it is not possible to identify the cause of the variability in prescriptive habits, but since it cannot be explained with differences in the epidemiology of the diseases among pediatric practice or with a different composition of patients, reducing variation among pediatricians should be a priority goal for health system.

Actions at the national and local level could be put in place to change prescribing patterns among physicians. At the national level, the most up-to-date guidelines on the use of ICSs should be disseminated and shared among relevant professional bodies, and training programs could be implemented that emphasize the importance of adherence among professionals.

At the regional level, dedicated training could be provided to the pediatrician to strengthen the relationship with parents and to constructively manage conversations with them when they expect a prescription that is inappropriate for the pediatric condition being treated. At the local level, finally, Audit and Feedback interventions could be implemented. Having the possibility to compare prescribing performance with peers could be an important opportunity for pediatricians to critically evaluate their ICS prescribing habits.

Some study limitations should be acknowledged. First, the present study took advantage of the availability of the regional health information systems, which provide data at patient level for all residents enrolled in the public health care service. It is worth noting that drug claims data are limited to refundable prescription drugs, and that all drugs of interest in this study are included. In some cases, drugs may be claimed paying out-of-pocket with a medical prescription, which would lead to an underestimation of drug use. However, especially in children, this practice is very unusual in Italy. Validation on the regional databases has been performed showing good performance [19] and health information systems of the Lazio Region are routinely used for studies in the context of drug utilization and pharmacoepidemiology [13]. Second, the reason for drug prescription is not available in our health information systems, therefore it is not possible to identify children who received drugs for asthma or wheezing and those with URTIs. A few differences exist in the criteria applied for the identification of potential asthmatic/recurrent wheezer children with those used by other authors [20], in particular concerning the number of drug prescriptions. Since there are no definitive criteria for using anti-asthmatic drug prescription as a proxy to identify asthmatic patients in preschool children, we considered the peculiarities of anti-asthmatic prescription profile in Italy, as reported in the Additional file 1. Following this approach, we decided to consider at least 3 prescriptions per year of salbutamol as a proxy for occurrence of more than 2 episodes of wheezing in a year, while up to 2 prescriptions/year of salbutamol are common in Italy for treatment of URTIs [3]. In the same way, we considered at least one prescription of long-acting beta-2-agonists, since it is extremely unlikely that a pediatrician could prescribe this kind of drugs to preschool children with no asthma or wheezing [21]. Third, results come from a single region in Italy and may be not generalizable to other geographic areas. However, the Lazio region has about 5 million residents, and our findings are in line with results of other studies carried out in different Italian regions [2–4, 6]. Furthermore, we did not directly analyze the role of parents in ICS prescribing decisions, because our information systems do not allow the linkage between the child and his parents.

### Conclusions

The SARS-CoV-2 outbreak indirectly led to a sharp decrease on the proportion of children exposed to ICS but had no effect on its variability among pediatric primary care providers.

The intra-regional drug prescribing variability underlines the lack of shared guidelines for appropriate ICS therapy in preschool children, and raises equity issues in access to optimal care.

Both LHD managers and individual pediatricians should be involved in training interventions, aimed to improve the rational use of ICSs, and reduce prescribing variation.

## Electronic supplementary material

Below is the link to the electronic supplementary material.

Supplementary Material 1

Supplementary Material 2

## Data Availability

The datasets used and analyzed during the current study are available from the corresponding author on reasonable request.

## List of Abbreviations

ATC: Anatomical Therapeutic Chemical
CI: Confidence Interval
DCR: Drug Claims Register
ECIS: Emergency Coronavirus Information System
EIS: Emergency Information System
HIS: Hospital Information System
ICS: Inhaled Corticosteroid
LHD: Local Health District
MOR: Median Odds Ratio
TEIS: Ticket Exemption Information System
URTI: Upper Respiratory Tract Infection
VC: Variance Component
