# Supplementary material for: Use of inhaled corticosteroids in preschool children and variability among pediatricians: a real-world analysis before and during the SARS-CoV-2 pandemic
Source: BMC Pediatr. 2023 Apr 1;23:151. doi: 10.1186/s12887-023-03968-5 (PMC10066959; doi:10.1186/s12887-023-03968-5)
Supplement: Supplementary file 1 — Supplementary Material 1 [file 12887_2023_3968_MOESM1_ESM.docx]

| **Clinical condition** | **Operational definition** | **Data source** |
| --- | --- | --- |
| 1. Asthma/recurrent wheezing:   diagnosis and proxies | At least one hospital discharge record or admission to emergency room with ICD-9-CM code of diagnosis: 493  *OR*  Exemption code number: 007.493  *OR* | HIS, EIS  TEIS |
|  | At least 3 prescriptions per year of salbutamol (ATC code: R03AC02), ipratropium bromid (R03BB01), salbutamol and ipratropium bromid (R03AL02), salbutamol and beclomethasone (R03AK13)  *OR*  At least 2 prescriptions per year of montelukast (R03DC03)  *OR*  At least 1 prescription per year of long-acting beta-2-agonists (R03AC1*), adrenergics in combination with corticosteroids or other drugs, excl. anticholinergics (R03AK*, excl. R03AK13). | DCR |
| b) Cystic fibrosis | At least one hospital discharge record or admission to emergency room with ICD-9-CM code of diagnosis: 277.0  *OR*  Exemption code number: 018. | HIS, EIS  TEIS |
| c) Immunodeficiency | At least one hospital discharge record or admission to emergency room with ICD-9-CM code of diagnosis: 042, V08, 279  *OR*  Exemption code number: 020. | HIS, EIS  TEIS |
| d) Malignant tumor | At least one hospital discharge record or admission to emergency room with ICD-9-CM code of diagnosis: 140-208, V10  *OR*  Exemption code number: 048. | HIS, EIS  TEIS |

**Additional file 1**

*The exclusion criteria*

EIS: Emergency Information System

DCR: Drug Claims Register

HIS: Hospital Information System

TEIS: Ticket Exemption Information System
