# Supplementary material for: Use of inhaled corticosteroids in preschool children and variability among pediatricians: a real-world analysis before and during the SARS-CoV-2 pandemic
Source: BMC Pediatr. 2023 Apr 1;23:151. doi: 10.1186/s12887-023-03968-5 (PMC10066959; doi:10.1186/s12887-023-03968-5)
Supplement: Supplementary file 2 — Supplementary Material 2 [file 12887_2023_3968_MOESM2_ESM.docx]

**Additional file 2**

*Sensitivity analysis:*

*Preschool children exposed to antidiabetic and antiepileptic drugs. Lazio, 2017-2020*
